# Balancing the fat: lipid droplets and human disease

**DOI:** 10.1002/emmm.201100671

**Published:** 2013-06-06

**Authors:** Natalie Krahmer, Robert V Farese, Tobias C Walther

**Affiliations:** 1 Department of Cell Biology, Yale School of MedicineNew Haven, CT, USA; 2 Gladstone Institutes, Departments of Medicine and Biochemistry & Biophysics, University of CaliforniaSan Francisco, CA, USA

**Keywords:** atherosclerosis, lipid droplet, lipodystrophy, metabolic syndrome, triglyceride storage

## Abstract

Lipid droplets (LDs) are dynamic, cytosolic lipid-storage organelles found in nearly all cell types. Too many or too few LDs during excess or deficient fat storage lead to many different human diseases. Recent insights into LD biology and LD protein functions shed new light on mechanisms underlying those metabolic pathologies. These findings will likely provide opportunities for treatment of diseases associated with too much or too little fat.

## Introduction

**Figure 1:**
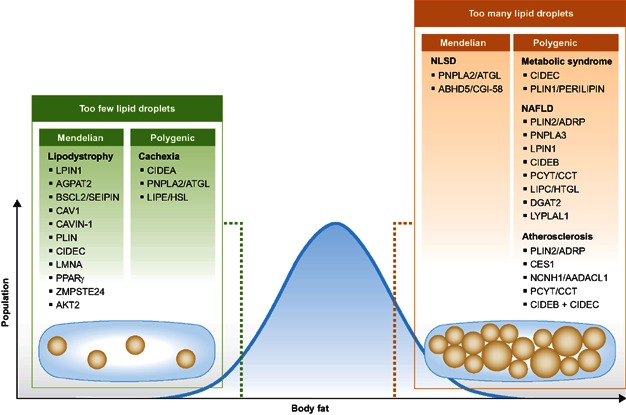
The balance of fat is important for human health Within the human population, too much or too little body fat results in different monogenic or polygenic pathologies. Human genes that are linked to these extremes of body fat and LD storage are shown and are discussed in the text.

Balancing fluctuations in availability and requirements of metabolic energy is important for life. With their high energy content, triglycerides (TGs) in cellular lipid droplets (LDs) are the largest energy reservoir in most organisms. Among different cell types, the plasticity of TG storage is impressive. However, saturating or exceeding the fat storage capacity leads to disease in humans. This is most obvious in obesity and linked pathologies. Less apparent is that deficiencies in forming or maintaining fat stores also are detrimental. While obesity and related diseases in most humans have multifactorial etiologies, recent discoveries in LD biology have begun to shed light on mechanisms that contribute to these metabolic diseases. Here, we examine metabolic disease from the LD viewpoint, reviewing the basic mechanisms that contribute to the development of pathologies associated with imbalances of LDs and fat storage (Fig 1).

### Lipid droplets: how cells store fat

To store excess lipids, such as sterols or fatty acids, cells esterify these lipids, forming neutral lipids, and package them into cytosolic LDs. In humans, adipocytes in white and brown adipose tissue are specialized for storing lipids in LDs. However, other cells also store lipids in LDs, including hepatocytes, enterocytes, macrophages and adrenocortical cells. Fat can also be prominent in tissues of blood formation or maturation, such as the bone marrow or thymus.

LDs have a nonpolar, neutral lipid core. In white adipocytes or macrophages, the core is mainly TG or sterol esters, respectively. In liver stellate cells, retinol esters are prominent. The LD core is surrounded by a phospholipid monolayer. In mammalian cells, the monolayer is mostly phosphatidylcholine with lesser amounts of phosphatidylethanolamine, phosphatidylinositol, lyso-phosphatidylcholine, and lyso-phosphatidylethanolamine (Liu et al, 2007). Phosphatidylcholine in particular is crucial as a surfactant to prevent LD coalescence, which might contribute to diseases of lipid storage excess. Depletion of proteins that change the phospholipid content dramatically changes LD morphology (Fei et al, 2011; Goodman et al, 2007; Guo et al, 2008; Krahmer et al, 2011).

Some proteins bind LD surfaces and regulate LD size and number. These include the PAT proteins [*e.g*., perilipin1, perilipin2/adipophilin (ADRP), perilipin3/Tip47, perilipin4/S3-12], CIDE (Cell Death Inducing DNA Fragmentation Factor) proteins, and several lipases. However, the LD proteome is much more complex: hundreds of proteins have been found in purified LDs fractions of different cell types, although many of these proteins may be contaminants from biochemical purifications (Martin et al, 2009). Recent quantitative proteomics approaches provide a means to distinguish *bona fide* LD proteins from contaminants with high confidence. In *Drosophila* cells, such an approach yielded ∼100 LD-enriched proteins (Krahmer et al, 2013).

LDs are dynamic organelles, constantly forming, growing or shrinking. They are mostly formed in the endoplasmic reticulum (ER), where enzymes catalyzing neutral lipid synthesis (*e.g*., acyl-CoA cholesterol acyltransferases (ACATs) for sterol esters and acyl CoA:diacylglycerol acyltransferases (DGATs) for TGs) predominantly reside (Farese et al, 2000, 2008). In fatty acid excess conditions, LDs rapidly increase their volumes, as seen for example in cell culture or murine small intestine. In part, this represents expansion of individual LDs by relocalization of TG synthesis to LD surfaces (Farese et al, 2009; Stone et al, 2009; Wilfling et al, 2013; Xu et al, 2012). During LD growth, the synthesis of neutral lipids and phospholipids is coordinated: as LD volume increases, surfaces expand, and phospholipids are needed to shield the neutral lipid core, reduce surface tension, and prevent LD coalescence (Krahmer et al, 2011). Thus, changes in LD phospholipid composition are important in determining their morphology and may play a role in diseases with altered lipid storage.

The main lipid storage tissue is the adipose tissue. In contrast to other cell types that form multiple small LDs with diameters of 100 nm to 5 µm, white adipocytes mainly form one giant, unilocular LD as large as 100 µm. These unilocular LDs likely provide the most efficient packaging of energy per volume. Several proteins such as the CIDE proteins were found to promote the formation of unilocular LDs.

LD degradation and mobilization of stored lipids are highly regulated processes (see Ahmadian et al, 2009; Lass et al, 2011). When fatty acids are required for energy generation or membrane lipid synthesis, lipids from LDs are used. In adipose tissue, catecholamines induce signalling pathways that activate adipose triglyceride lipase (ATGL) and hormone sensitive lipase (HSL), the lipases that act in concert on the LDs to degrade TGs. TGs may also be mobilized by lipases in autophagosomes (Czaja et al, 2009). Changes in lipolysis and lipid mobilization contribute to some lipid storage diseases.

LDs also function in multiple cellular processes, such as protein storage and degradation, viral replication and regulation of activities of associated enzymes (see Murphy, 2012; Thiele & Spandl, 2008; Walther & Farese, 2012).

**Glossary**

**ACAT 1**

Acyl-CoA:cholesterol acyltransferase 1 is one of two ER proteins that catalyze the formation of cholesterol esters by using cholesterol and fatty acyl CoA as substrates.

**Adiponutrin/PNPLA3**

Patatin-like phospholipase domain-containing protein 3 has TG lipase and acylglycerol *O*-acyltransferase activities.

**AGPAT2**

1-Acylglycerol-3-phosphate *O*-acyltransferase 2 catalyzes TG synthesis by using 1-acylglycerol-3 phosphate and fatty acyl CoA as substrates.

**AKT2**

AKT2, also called protein kinase B (PKB), is a serine/threonine protein kinase involved in insulin signalling.

**ATGL**

Adipose triglyceride lipase is a LD protein catalyzing the initial step in TG hydrolysis, the hydrolysis of TG into diacylglycerol.

**AZGP1**

Zinc-alpha-2-glycoprotein stimulates lipid degradation in adipocytes and causes the extensive fat losses associated with some advanced cancers. May bind polyunsaturated fatty acids.

**Caveolin 1**

Acts as a scaffolding protein within the specialized plasma membrane domains caveolae, and is involved in signalling.

**Cavin**

Structural protein of caveolae.

**CIDE proteins**

Cell Death Inducing DNA Fragmentation Factor proteins, are a family of LD proteins including three members CIDEA, CIDEB, CIDEC/FSP27. They were initially found to be involved in cell death. CIDEC, and possibly other CIDE proteins, promote LD growth by mediating directional TG transfer from smaller to larger LDs at LD contact sites.

**CGI-58**

Comparative gene identification-58, a coactivator of ATGL, which also interacts with perilipin1 and perlipin2/ADRP.

**CTα**

Also called CCT. CTP:phosphocholine cytidylyltransferase is the rate-limiting enzyme for phosphatidylcholine synthesis and catalyzes the reaction: CTP + phosphocholine → diphosphate + CDP-choline.

**DGAT1**

Acyl-CoA:diacylglycerol acyltransferase, one of two ER proteins catalyzing TG synthesis by using diacylglycerol and fatty acyl CoA as substrates.

**DGAT2**

Acyl-CoA:diacylglycerol acyltransferases, LD and ER protein catalyzing TG synthesis by using diacylglycerol and fatty acyl CoA as substrates.

**HSL**

Hormone sensitive lipase, a LD protein primarily catalyzing the second step in TG hydrolysis, the conversion of diacylglycerol into monoacylglycerol.

**LaminA**

Nuclear protein forming nuclear lamina to provide a framework for the nuclear envelope.

**Lipin1**

A phosphatidate phosphatase which catalyzes the conversion of phosphatidic acid to diacylglycerol for TG synthesis and localizes to the ER, nucleus and LDs.

**PAT proteins**

Family of LD proteins including Perilipin 1, Perilipin2/adipophilin (ADRP), Perilipin3/TIP47, Perilipin4/S3-12, Perilipin 5 (OXPAT). They interact with lipases and regulate their access to LDs. Moreover they are suggested to be involved in LD biogenesis and LD growth.

**Seipin/BSCL2**

Berardinelli–Seip congenital lipodystrophy type 2 protein localizes to ER–LD junctions and regulates neutral lipid storage. Its molecular function is unknown.

**TGH**

An ER luminal TG hydrolase.

**ZMPSTE24**

Zinc metallopeptidase STE24 homologue that activates laminA/C by proteolytic cleavage.

### Pathologies associated with too few lipid droplets

#### Genetic defects in lipid storage: lipodystrophies

Human lipodystrophies are the clinical manifestations of total (congenital generalized lipodystrophy, CGL) or partial (familial partial lipodystrophy, FPL) loss of body fat (see Garg & Agarwal, 2009; Huang-Doran et al, 2010; Vigouroux et al, 2011). Lipodystrophies cause severe changes of whole-body energy metabolism and are commonly associated with insulin resistance, hepatic steatosis, hypertension and other metabolic dysfunctions. Inability to store TGs in white adipose tissue gives rise to lipid storage in other tissues and tissue lipotoxicity. Lack of white adipose tissue leads to leptin deficiency and associated metabolic defects, such as insulin resistance. Many defects of severe lipodystrophies can be corrected by leptin supplementation (Oral et al, 2002; Shimomura et al, 1999).

Numerous gene defects cause CGL or FPL (reviewed in Garg, 2011). Here we review lipodystrophies with established connections to LD biology. Many of the lipodystrophy genes encode TG synthesis or storage proteins, and some act at the LD surface and are involved in LD formation and regulation. Still others regulate adipogenesis, thereby affecting LDs indirectly.

Two CGL loci encode proteins that regulate *de novo* TG synthesis: 1-acylglycerol-3-phosphate *O*-acyltransferase 2 (*AGPAT2*) and lipin1 (*LPIN1*). AGPAT2 catalyzes the formation of phosphatidic acid from lyso-phosphatidic acid and fatty acyl-CoA. Lipin1, a phosphatidic acid phosphatase, removes the phosphate group from phosphatidic acid to form diacylglycerol, the direct precursor of TG. *AGPAT2* mutations account for ∼50% of CGL (Van Maldergem et al, 2002). So far, none of the other AGPAT isoforms appears to be implicated in CGL. This suggests a specialized role for AGPAT2, at least in adipocytes. In contrast to AGPAT2, *LPIN1* mutations have been found to be associated with lipodystrophy only in mouse models; there are no known human *LPIN1* mutations causing lipodystrophy.

How *AGPAT2* and *LPIN1* mutations cause lipodystrophy is uncertain. Mutation of either enzyme causes reduced TG levels as well as accumulation of lipid synthesis intermediates, and phospholipids in tissues. For example, both *Lipin1* and *Agpat2* knockout mice have increased phosphatidic acid and lyso-phosphatidic acid levels in the adipose tissue (Gale et al, 2006; Peterfy et al, 2001). One model posits that lipid synthesis products, such as phosphatidic acid or lyso-phosphatidic acid, accumulate and inhibit adipocyte differentiation, perhaps by influencing PPARγ signalling (Gale et al, 2006; Yang et al, 2011).

Mutations of Berardinelli–Seip congenital lipodystrophy 2 gene (BSCL2/*SEIPIN*) also lead to severe CGL. *BSCL2* mutations in lipodystrophy patients result mostly in null alleles (Agarwal et al, 2004). *BSCL2* mutations exhibit more severe lipodystrophy and metabolic alterations than *AGPAT2* mutations (Van Maldergem et al, 2002).

How *BSCL2* deficiency causes lipodystrophy remains unclear. The *BSCL2* protein seipin is an ER protein, embedded in the membrane by a hairpin-type hydrophobic sequence. *BSCL2* is expressed in adipocytes and up-regulated during adipocyte differentiation in 3T3L1 cells (Chen et al, 2009). *BSCL2* knockdown inhibits adipocyte differentiation and suppresses PPARγ expression in this cell line (Chen et al, 2009). Moreover, it causes strong alterations in LD size, distribution, and number (usually fewer LDs) in multiple cell types and organisms, leading to markedly altered LD morphology (Fei et al, 2008; Szymanski et al, 2007; Tian et al, 2011). The yeast seipin orthologue forms oligomers and localizes near LDs (Binns et al, 2010; Lundin et al, 2006; Szymanski et al, 2007), suggesting a role in LD formation. However, seipin might also function in lipid biosynthesis pathways: its knockout alters cell phospholipids in yeast and *Drosophila* (Fei et al, 2011; Tian et al, 2011) and fatty acid composition in lymphoblastoid cell-lines, the latter from reduced desaturase activity (Boutet et al, 2009). In yeast and *Drosophila*, *seipin* knockout leads to accumulation of phosphatidic acid, which might induce LD fusion and alter LD morphology (Fei et al, 2011). Phosphatidic acid excess may also influence signalling pathways for adipocyte differentiation. In *Drosophila*, BSCL2/seipin depletion leads to loss of fat body lipids and to ectopic TG accumulation (Tian et al, 2011).

Deficiency of the membrane protein caveolin 1 also causes lipodystrophy. A homozygous nonsense mutation with complete loss of *CAVEOLIN1* (*CAV1*) expression causes CGL, and heterozygous mutations were found in patients with atypical partial lipodystrophy (Cao et al, 2008; Kim et al, 2008). Caveolin 1 is an essential organizer of caveolae, specialized cholesterol-rich microdomains in the plasma membrane that form invaginations. Caveolin 1 also localizes to LDs (Ostermeyer et al, 2001) and is highly expressed in adipocytes (Lisanti & Razani, 2001). A related protein, cavin 1, another component of caveolae (Hill et al, 2008), was found to be mutated in a patient with lipodystrophy (Matsuo et al, 2010; Shastry et al, 2010). Cavins interact with caveolin, and loss of cavin 1 leads to loss of caveolae and caveolin 1 mislocalization (Liu et al, 2008). Since mutations in *CAV1* and cavin 1 profoundly affect whole-body TG storage, caveolae may be important in lipid storage (Pilch & Liu, 2011). Caveolae are thought to function in the cellular response to mechanical stress, endocytosis, transcytosis, fatty acid uptake, LD formation, and lipid trafficking (Parton & Bastiani, 2010). The relationship between caveolae and LDs is still unclear.

Mutations in two LD proteins, perilipin1 and CIDEC, have been reported to cause FPL. A homozygous *CIDEC* mutation in a FPL patient led to a truncated protein that does not target LDs (Rubio-Cabezas et al, 2009) and cannot induce formation of unilocular LDs in adipocytes. Instead multiple small LDs form, similar to the murine knockout phenotype (Nishino et al, 2008). Two frame-shift mutations in the C-terminus of perilipin1 are associated with FPL and insulin resistance. Unlike wild-type perilipin1, the mutated perlipin1 fails to bind to and inhibit comparative gene identification-58 (CGI-58), leading to constitutive ATGL activation by CGI-58, resulting in increased basal lipolysis (Gandotra et al, 2011). While those mutations account for FPL in very few individuals, most FPL cases are caused by mutation of *lamin A* (*LMNA*). As a component of the nuclear lamina, *LMNA* is important for maintaining the nuclear envelope functions, loss of which leads to premature cell death and loss of adipocytes (Garg, 2004). This might also happen in FPL patients carrying mutations of *ZMPSTE24*, encoding a metalloprotease required for processing lamin A (Agarwal et al, 2003). In several other FPL patients, mutations in the genes for transcription factor *PPARγ* and *AKT2*, a factor involved in insulin signalling, were reported (George et al, 2004; Savage et al, 2003). Those mutations lead to defective adipocyte differentiation and thereby disturb TG storage. It is unclear why certain mutations cause FPL rather than CGL. Perhaps FPL is caused by deficient TG storage in existing adipocytes, and CGL is due to failure to form adipocytes.

In contrast to genetic inherited forms of lipodystrophies, acquired forms due to autoimmune diseases or drug treatment are much more common (see Garg, 2004). Protease inhibitors used to treat HIV infection are the most frequent cause of acquired lipodystrophy. The mechanism of the pathogenesis is not well understood. HIV protease inhibitors change the expression and localization of transcription factors, such as PPARγ or SREBP, which mediate adipocyte differentiation (Caron et al, 2001). Moreover, they increase basal and stimulated lipolysis in adipocytes, as shown in 3T3-L1 cells. This effect is mediated by a decrease in perilipin levels on the LDs by increased lysosomal perilipin degradation (Adler-Wailes et al, 2005).

#### Rapid loss of triglyceride stores: cancer cachexia

Cachexia, a complex metabolic syndrome, is common in cancer patients, particularly gastrointestinal, prostate and lung cancer (Tisdale, 2005). Unlike lipodystrophy, which features chronic deficiency of adipose tissue, cachexia is an acute wasting disease. Lipid metabolism is fundamentally changed, leading to dramatically reduced body weight, caused early by a loss of adipose tissue and later by atrophy of skeletal muscle (Bing & Trayhurn, 2009). Those changes are associated with poor response to chemotherapy and high mortality: 15–20% of cancer deaths are caused by cachexia (Tisdale, 2002). Increased lipolysis is a key factor in cachexia, and cachexia patients show elevated blood glycerol and fatty acids (Shaw & Wolfe, 1987).

The role of LDs in cachexia is beginning to be unravelled. Recent advances reveal that ATGL, and not HSL as previously thought, mediates increased lipolysis in cachexia (Das et al, 2011). *Atgl* knockout mice are completely protected from adipose tissue wasting and had no increased lipolysis after inducing cachexia, despite high levels of lipid-mobilizing factors, such as zinc-alpha-2-glycoprotein 1 (AZGP1), tumour necrosis factor α (TNF-α), or interleukin-1 (Das et al, 2011). Thus, inflammatory and lipolytic mediators that activate ATGL, potentially secreted by the tumour, might cause uncontrolled loss of adipose tissue in cachexia. Intriguingly, skeletal muscle loss was also absent in *Atgl* knockout mice.

### Pathologies associated with too many lipid droplets

An increasingly sedentary lifestyle and unhealthy eating habits have made obesity and its associated pathologies a dramatic global health issue. These diseases are characterized by over-accumulation of TGs and LDs. Additionally, some genetic diseases are associated with over-accumulation of LDs. Understanding these rare conditions might provide therapeutic insights for their cure and uncover new obesity treatments. Here, we review molecular insights provided by these more rare conditions.

#### Monogenetic diseases of triglyceride and lipid droplet accumulation

Neutral lipid storage disease (NLSD) is a rare, autosomal recessive disorder characterized by substantial systemic TG accumulation (Schweiger et al, 2009). Mutations in genes encoding adipose triglyceride lipase (ATGL) and its cofactor CGI-58 cause NLSD. Although these proteins collaborate during lipolysis, mutations in each cause different symptoms. Mutations in *CGI-58* are associated with ichthyosis, a permeability barrier defect of the skin; ATGL mutations cause severe cardiomyopathy and systemic lipid accumulation in humans and mice (Lefevre et al, 2001; Schweiger et al, 2009). The differences suggest that CGI-58 has other, ATGL-independent functions.

New insights into the molecular mechanism of cardiomyopathy in *ATGL*-deficient NLSD reveal a role for LDs in cellular signalling (Haemmerle et al, 2011). Hydrolysis of TGs from LDs by ATGL releases activators of the PPARα/peroxisome proliferator-activated receptor-γ coactivator 1 (PGC1) complex in cardiac myocytes. Activity of the complex increases with fatty acid supplies in the cell and regulates mitochondrial oxidative capacity. Cardiac ATGL deficiency decreases PGC-1 expression, leading to mitochondrial dysfunction and lipid accumulation in the heart, thus causing heart failure. Treatment of *Atgl* KO mice with pharmaceutical PPARα agonists reverses mitochondrial dysfunction and restores heart function. Which LD proteins are involved in regulating lipolysis for PPARα signalling and which specific products of the ATGL reaction mediate PPARα-PGC1 activation remain unknown. Nonetheless, these findings suggest PPARα agonists will be useful in treating patients with NLSD cardiomyopathy.

#### Lipid droplets in obesity and associated diseases

Adipose tissue is critical in regulating lipid and glucose metabolism. It buffers lipid excess by sequestering fatty acids into TGs, thereby protecting the body from lipotoxicity, and releases fatty acids and glycerol for peripheral tissues during starvation. Adipose tissue also exerts an important endocrine function by secreting factors, such as leptin and adiponectin that regulate insulin sensitivity or inflammation (Capeau et al, 2011; Friedman, 2009). Obesity can lead to altered systemic metabolism, including the metabolic syndrome, which increases the risk of type II diabetes, steatohepatitis and coronary heart disease. With adipocyte hypertrophy, insufficient amounts of adipokines are secreted to maintain insulin sensitivity (Hotamisligil, 2003), and pro-inflammatory cytokines, such as monocyte chemoattractant protein-1 (MCP-1) and TNF-α, are secreted that induce macrophage infiltration and inflammation (Hotamisligil et al, 1993). TNF-α increases lipolysis and down-regulates proteins that promote TG storage and protect LDs from lipolysis (Guilherme et al, 2008). TNF-α reduces cellular perilipin levels, likely leading to increased basal lipolysis and free fatty acid levels in the blood (Souza et al, 1998a). These insulin resistance-promoting effects of TNF-α can be antagonized by anti-diabetic agents, such as thiazolidinediones (Souza et al, 1998b).

Normally, the adipose tissue sequesters free fatty acids and other lipids in form of inert TGs. However, under certain conditions, such as the metabolic syndrome, the neutral lipid storage capacity of the adipose tissue is exceeded. Free fatty acids flow from adipose to peripheral tissues, and ectopic lipids accumulate in skeletal muscle, heart or liver (van Herpen & Schrauwen-Hinderling, 2008). Excess bioactive lipids, such as diacylglycerol, free fatty acids, and related derivatives, may cause lipotoxicity, with lipids interfering with signalling pathways and promoting insulin resistance in skeletal muscle and hepatic tissue (Schaffer, 2003; Virtue & Vidal-Puig, 2010). Usually, there is also accompanying leptin resistance, which promotes insulin resistance (El-Haschimi et al, 2000).

Although multiple genetic and environmental factors contribute to the development of the metabolic syndrome, at the cellular level TG accumulation equates with massive over-accumulation of LDs in adipose and other tissues. LD-associated proteins in adipose tissue can in turn influence the pathology of excess TG storage in some individuals. Proteins, such as FSP27/CIDEC and perilipin1, are crucial for unilocular LDs in adipose tissue. For example, FSP27/CIDEC influences the development of the metabolic syndrome by regulating TG storage in adipocytes. *FSP27*/*CIDEC* polymorphisms influence obesity risk (Dahlman et al, 2005; Zhang et al, 2008, 2011). Studies examining expression of CIDE proteins and perilipin as a function of insulin sensitivity found that the levels of mRNAs that encode these LD-associated proteins correlate inversely with insulin sensitivity subjects similar body mass index (Guilherme et al, 2008). This indicates that high levels of expression of these TG storage-promoting proteins might help to sequester lipids in the adipose tissue and to protect against insulin resistance. However, paradoxically, *FSP27*/*CIDEC* knockout mice exhibit increased energy expenditure and are protected from diet-induced obesity and insulin resistance (Nishino et al, 2008). All three members of the CIDE family (CIDEA, CIDEB, FSP27/CIDEC) localize to LDs via their C-terminal CIDE-domain, as shown in different cell lines (Gong et al, 2009). In adipocytes, FSP27/CIDEC is important in forming unilocular LDs. Depleting FSP27/CIDEC prevents their formation, and overexpressing it in other cell types induces larger and fewer LDs (Gong et al, 2011; Jambunathan et al, 2011; Nian et al, 2010; Nishino et al, 2008; Puri et al, 2007). FSP27/CIDEC localizes mainly to contact sites between LDs, where it seems to facilitate lipid transfer from smaller to larger LDs in 3T3L1 cells (Gong et al, 2011).

Perilipin1 also regulates TG storage in white adipocytes (Greenberg et al, 2011). *Perilipin1* knockout mice are lean and protected from diet-induced obesity (Martinez-Botas et al, 2000; Tansey et al, 2001). Perilipin1 localizes to LD surfaces and is an important regulator of lipolysis, protecting LDs from basal lipolysis (Zhai et al, 2010). However, when lipolysis is stimulated, perilipin1 regulates access of lipases to LDs (Sztalyrd et al, 2003).

In humans, expression of *PERILIPIN* and *FSP27*/*CIDEC* in adipose tissue inversely correlates with insulin resistance (Puri et al, 2008), and insulin-sensitive obese individuals have higher levels of perilipin1 and CIDEC in adipose tissue than insulin-resistant subjects of the same weight, suggesting that higher expression of these proteins promotes TG storage in adipose tissue and protects from lipotoxicity. These findings in humans contrast with findings in mice, in which the lack of *perilipin1* or *FSP27* leads to increased insulin sensitivity (Martinez-Botas et al, 2000; Nishino et al, 2008; Tansey et al, 2001). Such findings highlight the difficulties in extrapolating results from experiments in mice to human pathologies.

#### Lipid droplets in hepatic steatosis and liver disease

The metabolic syndrome is often accompanied by nonalcoholic fatty liver disease (NAFLD), the accumulation of TG-containing LDs in hepatocytes. Several studies show that during steatogenenesis, the expression pattern of several LD-associated PAT proteins changes in a PPARγ-dependent manner (Inoue et al, 2005; Matsusue et al, 2008; Schadinger et al, 2005). Notably, perilipin1, normally only in adipose tissue, is expressed in human hepatocytes of fatty liver (Fujii et al, 2008; Straub et al, 2008). Also ADRP levels are up-regulated in steatosis in humans and in mice (Motomura et al, 2006). ADRP-deficient mice are resistant to diet-induced fatty liver, implicating ADRP in hepatic lipid accumulation (Chang et al, 2006).

A polymorphism of another LD protein PNPLA3/adiponutrin was linked to increased risk for NAFLD development (Romeo et al, 2010). The molecular mechanism underlying PNPLA3/adiponutrin function in hepatic lipid metabolism is controversial. A recent study reported LPA acyltransferase activity for PNPLA3/adiponutrin (Kumari et al, 2012) in contrast to other studies detecting TG hydrolase activity (Jenkins et al, 2004). The mutation associated with increased risk for NAFLD was characterized as gain of function mutation leading to TG accumulation and thereby explaining the increase in NAFLD disposition (Kumari et al, 2012; Li et al, 2012).

Genetic variants in genes encoding two proteins with TG lipase activity, hepatic lipase (*LIPC*/*HTGL*) (Yamada et al, 2011) and lysophospholipase-like1 (*LYPLAL1*) (Speliotes et al, 2011), and the DGAT2 enzyme involved in TG synthesis (Kantartzis et al, 2009) have also been associated with the risk of developing hepatic steatosis. Interestingly the reported genetic variants were only associated with increased risk for hepatic steatosis but not insulin resistance (see Farese et al, 2012), consistent with the paradigm that sequestration of lipids into liver TG might protect from free fatty acid-induced lipotoxicity that promotes insulin resistance.

The liver secretes TG-rich very low-density lipoproteins (VLDL) for distributing lipids throughout the body. Blocking VLDL secretion leads to NAFLD but not always insulin resistance (Sun & Lazar, 2013). Tissue-specific ablation of hepatic CTP:phosphocholine cytidylyltransferase α (CTα), the rate-limiting step in phosphatidylcholine synthesis, blocks VLDL secretion and leads to hepatic steatosis in mice (Jacobs et al, 2004). CTα targeting to LDs, required for LD expansion (Krahmer et al, 2011), suggests that loss of this capability contributes to the phenotype. However, how cytosolic LDs are coordinated with VLDL secretion is not understood. This complex process might be regulated by the coordinated action of multiple proteins. Liver TG hydrolase (TGH) might play an important role in mobilizing TG from LD for VLDL secretion as ectopic TGH expression leads to increased VLDL secretion and a reduction of cellular TG pools (Lehner & Vance, 1999). However, as TGH is an ER enzyme with its active site localizing to the ER lumen (Lehner et al, 1999), it remains enigmatic how TGH accesses TGs in the LD core. The expression and compartmentalization of lipin may also be important for the regulation of VLDL formation. TGs synthesized by expression of lipin-1 were reported to be mainly channelled into VLDL in McA-RH7777 cells (Khalil et al, 2009). CIDEB is localized to ER and LDs, interacts with apoB, and promotes TG secretion in VLDL in murine hepatocytes (Ye et al, 2009). CIDEB, by interacting with LDs and the ER at the cytosolic face, might channel TGs stored in cytosolic LDs into VLDL for secretion.

Hepatitis C virus (HCV) infection also strongly increases the risk for hepatic steatosis by inducing metabolic changes in infected hepatocytes. The HCV core protein is targeted to LDs by a DGAT1-dependent mechanism (Herker et al, 2010), and LDs are important for the assembly of infectious viral particles (Miyanari et al, 2007). LD targeting of HCV core induces clustering and cellular redistribution of LDs in cultured cells (Boulant et al, 2008). The expression of a genetic variant of core is sufficient to induce steatosis in transgenic mice (Moriya et al, 1997). Although numerous mechanisms might contribute to steatosis caused by HCV core (Herker & Ott, 2011), at least in part HCV induces LD accumulation by inhibiting lipolysis and stabilizing LDs (Harris et al, 2011). This induction of steatosis also apparently depends on DGAT1.

#### Lipid droplets in cardiovascular disease

Atherosclerosis is a leading cause of death in industrial countries. Accumulation of cholesterol esters in arteries is tightly linked to atherosclerosis and can lead to myocardial infarction, stroke, or sudden cardiac death. Cholesterol esters in arteries are stored mainly in LDs of macrophage foam cells, named for their appearance by microscopy Atherogenic apoB-containing lipoproteins (low density lipoprotein (LDL), chylomicron and VLDL remnants), containing large amounts of cholesterol, accumulate in the subendothelial space and are taken up by macrophages (Moore & Tabas, 2011). In macrophages, internalized lipoproteins are hydrolyzed, and the free cholesterol is re-esterified by ACAT enzymes, in particular ACAT1 in macrophages, to cholesterol esters for storage in LDs. The specific protein composition of cholesterol ester droplets is unknown, and besides ACAT1, specific factors for forming and regulating cholesterol esters LDs are unknown. Recently, specific PAT proteins were shown preferentially on TG- or cholesterol ester-containing LDs in different cell lines (Hsieh et al, 2012), indicating that LD protein composition might vary depending on the neutral lipid content of the LD.

Macrophages that accumulate large amounts of cholesterol esters become foam cells. Cholesterol esterification in foam cells seems to be protective, since free cholesterol is toxic to cells and pro-inflammatory (Kellner-Weibel et al, 1998). Cholesterol in LDs continuously undergoes a cycle of cholesterol ester hydrolysis and re-esterification (Brown et al, 1980). Free cholesterol effluxes from macrophages to extracellular acceptors, such as nascent high-density lipoprotein (HDL) and apolipoprotein-AI, which mediate reverse cholesterol transport from peripheral tissues to liver and are inversely correlated with atherosclerosis (Khera et al, 2011). Thus, cholesterol ester formation and storage in macrophages likely provide buffering capacity until cholesterol can be removed from the arterial wall by cholesterol efflux and reverse cholesterol transport (Moore & Tabas, 2011). Consistent with this notion, ACAT1 knockout in macrophages of hyperlipidemic mice leads to a pro-inflammatory state in skin and, if anything, increased atherosclerosis (Accad et al, 2000; Fazio et al, 2001; Su et al, 2005). If cholesterol accumulation overwhelms removal mechanisms, inflammation and pathology progress, similar to that of a wound, leading to plaque formation, rupture and thrombosis (Bornfeldt & Tabas, 2011).

Several LD proteins (*e.g*., ADRP, CIDEB and CIDEC) are up-regulated in foam cells and may promote storage of cholesterol esters in LDs, thereby protecting from cellular toxicity from free cholesterol (Yuan et al, 2012). However, peritoneal macrophages from *Adrp* knockout mice accumulate fewer LDs upon cholesterol loading, and in atherosclerosis-prone *apoE* knockout mice, ADRP depletion decreases the number of LDs in foam cells, yet protects mice from atherosclerosis (Paul et al, 2008).

For cholesterol efflux, the rate-limiting step is cholesterol ester hydrolysis from LDs. Different lipases may act on the LD surface to mobilize cholesterol (Hua et al, 2009). HSL was thought to provide most of cholesterol ester hydrolase activity, but deleting HSL does not reduce cholesterol ester hydrolysis (Osuga et al, 2000). Other candidates include CES1 (carboxylesterase 1), which decreases cholesterol ester storage in human macrophages and promotes cholesterol efflux (Ghosh et al, 2003). Its expression in macrophages reduces atherosclerosis in LDL-receptor deficient mice (Zhao et al, 2007). Arylacetamide deacetylase-like 1 (AADACL1) depletion increases atherosclerosis and cholesterol ester storage in murine macrophages (Sekiya et al, 2009), and its overexpression in THP-1 cells reduces cholesterol ester storage (Igarashi et al, 2010). However, CES1 and AADACL1 localize to the ER with their active sites facing the lumen and not the LD surface (Quiroga & Lehner, 2011), indicating they likely function in cholesterol ester hydrolysis at a different site from LDs. Other lipases might be responsible for cholesterol ester mobilization from macrophage LDs.

Besides cytosolic cholesterol ester hydrolysis on the LD surface, cholesterol can be mobilized from LDs by autophagy, leading to degradation of cholesterol ester by lysosomal acid lipases (Marcel et al, 2011). The released free cholesterol provides a major source of ABC-AI-dependent cholesterol efflux to apoAI and a minor proportion of HDL-mediated efflux. Macrophages from autophagy-deficient *autophagy protein-5* (*Atg5*) knockout mice show a reduced cholesterol efflux (Marcel et al, 2011). In foam cells, autophagy seems to be activated in response to cholesterol loading. These findings suggest that cholesterol ester accumulation in macrophages triggers autophagy and that accelerators of this pathway slow atherosclerosis.

In addition to esterifying excess cholesterol, macrophages up-regulate phosphatidylcholine synthesis to protect against cytotoxicity (Shiratori et al, 1994). Such up-regulation is induced in *Drosophila* cells and mammalian macrophages, at least in part, by relocalizing the rate-limiting enzyme choline–phosphate cytidylyltransferase to membranes and LDs (Krahmer et al, 2011), thereby increasing enzyme activity. Posttranslational regulation leads to increased phosphatidylcholine synthesis, helping to maintain a constant phosphatidylcholine:free cholesterol ratio in the cell membrane and preventing free cholesterol toxicity (Tabas, 2000). This mechanism might help to adapt cellular phosphatidylcholine synthesis to elevated needs during LD formation. Macrophages from CTα knockout mice die faster under cholesterol loading (Zhang et al, 2000). Macrophage cell death is an important factor in the progression of unstable plaques, and thus, phospholipid synthesis pathways allow intervention to prevent advanced lesions.

## Summary and outlook

The balance of fat is important for human health. Too much or too little is linked to common disease pathologies. LDs store fat, but despite their importance for cell and organismal physiology, relatively little is known about many basic processes in LDs in different tissues. Identification of key players in LD biology will uncover molecular processes underlying lipid storage and promote better understanding of LD-linked diseases. Important proteins regulating lipid storage have been recently identified, and a number of genes encoding LD proteins have been linked to metabolic diseases. Some of these molecular insights could lead to therapies. For example, ATGL inhibitors could offer the possibility of treating cachexia, or DGAT1 inhibitors could be tested as a means to prevent hepatitis C infection. The recent upsurge in LD biology research will lead to more knowledge of lipid storage and novel molecular-based approaches for treating diseases due to over- or under-storage of fat.

**Pending Issues**

What are the physiological functions of different LD populations, *e.g*. generated by different enzymes and containing different lipids? Do specific factors regulate their formation and consumption?

Why do mutations in some lipodystrophy genes cause congenital generalized lipodystrophy and others familial partial lipodystrophy?

What role does altered phospholipid levels play in the pathogenesis of lipodystrophies in patients with *AGPAT2* and *BSCL2*/*SEIPIN* mutations? What is the mechanism of action for these proteins?

Which factors related to ATGL-mediated lipolysis trigger cancer cachexia?

What determines the capacity of cells to store neutral lipids? What are the mechanisms of lipotoxicity leading to atherosclerosis, insulin resistance, and other pathologies?

How do FSP27/CIDEC and PAT proteins influence insulin sensitivity? Why are *CIDE* knockout mice protected from obesity and insulin resistance whereas in humans, expression levels inversely correlate with insulin sensitivity?

How are neutral lipid storage in LDs and neutral lipid secretion via VLDL balanced and regulated? Which proteins regulate those processes and which enzymes mobilize neutral lipids for secretion? Is there the possibility for pharmacological intervention for the treatment of NAFLD and its progression to NASH?
